# Adaptive Managers as Emerging Leaders During the COVID-19 Crisis

**DOI:** 10.3389/fpsyg.2021.661628

**Published:** 2021-04-13

**Authors:** Abdulah Bajaba, Saleh Bajaba, Mohammad Algarni, Abdulrahman Basahal, Sarah Basahel

**Affiliations:** ^1^Department of Management, Louisiana Tech University, Ruston, LA, United States; ^2^Department of Business Administration, King Abdulaziz University, Jeddah, Saudi Arabia; ^3^Department of Human Resource Management, King Abdulaziz University, Jeddah, Saudi Arabia; ^4^Department of Management Information Systems, King Abdulaziz University, Jeddah, Saudi Arabia

**Keywords:** adaptive personality, adaptive performance, COVID-19, self-efficacy, motivation

## Abstract

The coronavirus disease 2019 (COVID-19) has taken the world by surprise and has impacted the lives of many, including the business sector and its stakeholders. Although studies investigating the impact of COVID-19 on the organizational structure, job design, and employee well-being have been on the rise, fewer studies examined the role of leadership and what it takes to be an effective leader during such times. This study integrates social cognitive theory and conservation of resources theory to argue for the importance of adaptive personality in the emergence of effective leaders during crisis times, utilizing the crisis of COVID-19 as the context for the study. We argue that managers with an adaptive personality tend to have increased self-efficacy levels to lead during a crisis, resulting in increased motivation to lead during the COVID-19 crisis. Furthermore, managers with increased motivation to lead during the COVID-19 crisis are argued to have enhanced adaptive performance, thereby suggesting a serial mediation model where crisis leader self-efficacy and motivation to lead during the COVID-19 crisis act as explanatory mechanisms of the relationship between the adaptive personality and performance of the manager. In order to test our hypotheses, we collected data from 116 full-time managers in Saudi Arabia during the COVID-19 crisis and used hierarchical linear regression as the method of analysis. The findings support all of the hypotheses. A discussion of the results, contributions, limitations, and future directions is included.

## Introduction

Since the initial widespread of coronavirus disease 2019 (COVID-19) beginning in early 2020, the world has been experiencing unprecedented times of disruptions and disorder ranging from economic losses, unemployment, and organizational and job-design overhauls all the way to health issues and increased mortality rates (Chong et al., 2020; Gallup, 2020; Guyot and Sawhill, 2020). According to the World Health Organization (2020), as of December 28, 2020, there has been a total number of 79,673,754 confirmed cases of COVID-19, including 1,761,381 deaths. Numerous studies have attempted to examine the impact of such a pandemic on the workplace and the employees (e.g., Caldas et al., 2020; Trougakos et al., 2020). For instance, a recent study by Fu et al. (2021) found that the anxiety levels of the employees are affected by the reported number of COVID-19 cases and the acceleration and velocity at which the reported number is changing, thus affecting the employees' work functioning (engagement, performance, and emotional exhaustion). *However, fewer studies have looked at what constitutes effective leadership in the workplace during such a crisis and its potential antecedents* (e.g., Hu et al., 2020; Yuan et al., 2021).

Leaders play an important role in the workplace due to their capacity to influence the environment by providing employees with the necessary resources to overcome their job demands or mitigating potential resource loss (Bakker and Demerouti, 2017). For instance, a study by Fernet et al. (2015) found that transformational leadership is related to fewer follower job demands (e.g., emotional, physical, and cognitive demands) and increased job resources (e.g., quality of relationships, participation in decision-making, and job recognition), which indirectly lead to the followers having positive work attitudes and increased job performance. As a result, having an effective leader is especially crucial in times of massive resource loss and increased demands, such as the case with the COVID-19 crisis. Due to the unexpected and disorderly nature of crises, having flexibility and readiness to change as a manager is of utmost importance as such circumstances are characterized by constrained rationality, ambiguity, time pressure, and life and death stakes (Parry, 1990; Mumford et al., 2007; Sommer et al., 2016). In other words, managers who demonstrate adaptive performance (i.e., effective handling of emergencies and work stress, creative problem solving, constant learning, and interpersonal adaptability; Pulakos et al., 2000) are necessary to provide the most suitable resources and adjust the department/team's structure, job design, and targets in order to coincide with the COVID-19 crisis.

According to the social cognitive theory (SCT; Bandura, 1986), the personal factors of the individual (i.e., cognitive, affective, and biological factors) affect their behavioral patterns. Therefore, a manager who demonstrates adaptive performance is more likely to have an adaptive personality. Although the concept of adaptivity has been thoroughly discussed in the literature (Judge et al., 1999; Kilcullen, 2004; Ployhart and Bliese, 2006; Hirschi et al., 2015; Rudolph et al., 2017), there is little research that has conceptualized it as a personality trait rather than a skill, motivation, or capacity. A recent study by Fuller et al. (2018) operationalized the concept of adaptive personality and defined it as “a predisposed willingness to change oneself in response to the needs and demands of a change in the environment. Individuals with adaptive personalities focus upon maintaining a good fit with their environment, so they are mindful of changes that occur and are ready to modify thought and behavior patterns to accommodate the new situation” (p. 12). Those with adaptive personalities tend to be calm during stressful situations and possess the personal resources needed to confidently embrace change and make the best out of it (Fuller et al., 2018). *Scholars call for research that empirically validates constructs of adaptivity as a personal trait* (Baard et al., 2014).

In addition to emphasizing the role of personal factors in influencing the individual's behaviors, SCT also sheds light on the critical role of self-efficacy. Self-efficacy refers to the belief the individual holds regarding their capability to achieve the desired results (Bandura, 1999). It is based on the level of self-efficacy that individuals choose which challenges to undertake and how much energy to invest in overcoming them (Locke and Latham, 1990; Bandura, 1991). One such form of self-efficacy is the leaders' efficacy to lead during a crisis (i.e., crisis leader self-efficacy; Hadley et al., 2011). According to the Conservation of Resources (COR) theory, people are motivated to obtain, retain, and protect their resources (Hobfoll, 1989). As a result, individuals are more likely to be motivated to take on opportunities for resource gain or protection from resource loss when they perceive they can do so (resource investment principle; Hobfoll et al., 2018). *In the context of crisis management*, Hadley et al. (2011) *call for research by proposing a theoretical framework in which crisis leader self-efficacy and motivation to lead during a crisis serve as two explanatory mechanisms of the relationship between the leader's characteristics and performance during a crisis*.

**Figure 1:**
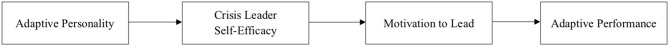
Hypothesized model. Motivation to lead and adaptive performance reflect the levels of motivation and performance during the COVID-19 crisis, respectively.

This study attempts to answer the mentioned calls for research and gaps by integrating SCT and COR theory in the context of the COVID-19 crisis, thereby offering multiple contributions to the crisis management literature (James et al., 2011). First, this study examines three antecedents of effective leadership during the COVID-19 crisis (i.e., leader adaptive performance). Second, it extends previous literature arguing that personality plays a role in predicting adaptive performance by empirically testing a newly developed measure of adaptive personality utilizing a sample of full-time managers in Saudi Arabia during the COVID-19 crisis (Huang et al., 2014; Park and Park, 2019). Third, the study examines crisis leader self-efficacy and motivation to lead during the COVID-19 crisis as two explanatory mechanisms through which the manager's adaptive personality affects his/her adaptive performance during the pandemic (see Figure 1).

## Theoretical Background

Introduced by Bandura (1986), SCT is a learning theory that states that individuals acquire new behaviors through observational learning and that the individuals' personal factors, the behavior itself, and the environment affect and are affected by each other, a concept known as triadic reciprocal causation (Bandura, 1999). Unlike other social learning theories, SCT emphasizes the role of personal agency such that people are producers as well as products of their environment (Bandura, 1999). Simply put, people are self-reactors who are able to motivate, regulate, and guide their behaviors instead of solely being controlled/shaped by the imposed environment. Perceived self-efficacy is considered as one of the core self-regulatory mechanisms through which someone is motivated to engage in a certain behavior or not (Bandura, 1999)—having the belief that an individual is able to produce the desired results influences their decision-making, perception of threats and challenges, and vulnerability to the imposed environment (Bandura, 1999). More specifically, those with high self-efficacy tend to be more motivated to engage in behaviors that enhance their well-being, provide them with more resources, and/or protect their current ones (Hobfoll et al., 2018).

The COR theory, a motivational theory introduced by Hobfoll (1989), defines resources as “those objects, personal characteristics, conditions, or energies that are valued by the individual or that serve as a means for the attainment of these objects, personal characteristics, conditions, or energies” (p. 516). Although resources are usually thought of in terms of money, time, or objects, Hobfoll (1989) emphasizes the importance of personal characteristics, such as personality traits and skills, as invaluable resources in dealing with stressors. According to the COR theory, stressful situations are characterized by (1) perceived threat toward one's current resources, (2) loss of one's current resources, and/or (3) failure to gain additional resources following significant effort or investment (Hobfoll, 1989; Hobfoll et al., 2018). When individuals are faced with such stressful situations, they tend to act in one of two ways depending on their current pool of resources. If the individual has the necessary resources to deal with the stressful situation, they tend to utilize their current pool of resources to offset the resource loss. Moreover, if the stressful situation imposes circumstances of huge resource loss, such as in the case of a crisis, individuals are more likely to utilize their resources to also gain additional resources in the process as resource gains become more salient/important in such contexts (gain paradox principle; Hobfoll, 1989; Hobfoll et al., 2018). On the other hand, if the individual lacks the necessary resources to deal with the stressful situation, they tend to be more vulnerable to it and enter a defensive, aggressive, and potentially irrational state by engaging in behaviors of withdrawal or self-protection as a last resort (desperation principle; Hobfoll, 1989; Hobfoll et al., 2018). The drawn insights from SCT and COR theory can be illustrated in the type of behaviors managers engage in when dealing with stressful situations in the workplace, such as those of a crisis.

In their theoretical framework of leader development and performance, Chan and Drasgow (2001) discussed the concepts of self-efficacy and personal resources as two important characteristics in influencing a leader's motivation to lead in a certain context and their performance as a result. Hadley et al. (2011) built upon the work of Chan and Drasgow (2001) and apply it to the context of crisis; more specifically, they introduce and develop a measure for the concept of crisis leader self-efficacy, which refers to the efficacy beliefs the leader holds about themselves regarding information assessment and decision-making in public health and safety crisis. The information assessment aspect of crisis leader self-efficacy involves the leader's beliefs regarding their capability to determine the flow of information during a crisis, collect and identify data needed for crisis resolution, and prevent/reduce errors and biases (Hadley et al., 2011, p. 634). On the other hand, crisis decision-making involves the leader's beliefs regarding their capability to generate response options and utilize the gathered data to evaluate, recommend, and choose the best course of action during a crisis (Hadley et al., 2011, p. 634). The authors argue that leaders with high self-efficacy to lead in a crisis are more likely to be motivated to lead and perform better during a crisis. Furthermore, they argue that crisis leader self-efficacy can be predicted by the leader's characteristics, such as individual differences, general leadership background, crisis training, and procedural preparedness (Hadley et al., 2011).

### Adaptive Personality and Crisis Leader Self-Efficacy

Although the positive impact of proactive personality regarding self-initiated constructive change has been thoroughly discussed in the literature (Fuller and Marler, 2009; Spitzmuller et al., 2015), adaptivity is considered as a crucial, initial step when faced with situations requiring organizational change (Strauss et al., 2015), such as in the case of the COVID-19 crisis. Strauss et al. (2015) argue that adaptivity is crucial for subsequent proactivity as it creates critical resources during instances of organizational change by acquiring knowledge of and adjusting to changes in stakeholders' goals and strategy, enhancing one's self-efficacy to cope with such change, and maintaining positive relationships. Fuller et al. (2018) distinguish adaptive personality from proactive personality and their polar opposites by proposing the Change-Control Circumplex Model, which is based on two axes: control orientation and change orientation. Whereas control orientation refers to the tendency to the preference to feel in control of one's changing environment (Rothbaum et al., 1982), change orientation refers to the tendency to approach or avoid change (Fuller et al., 2018). Although both proactive and adaptive personalities are characterized by approaching change, proactive personality emphasizes primary control (change the environment to fit one's needs) while adaptive personality emphasizes secondary control (accommodate to environmental conditions; Fuller et al., 2018). Adaptive individuals tend to be present-oriented, flexible, quick learners, optimistic regarding change, and willing to accommodate change and try better ways of doing things (Fuller et al., 2018).

Drawing insight from SCT, we argue that the flexible and accommodating nature of adaptive personality regarding embracing change is more likely to welcome numerous experiences of adaptation as imposed by the environment during the individual's lifetime (Bandura, 1986). Namely, instead of resisting change, such as in the case of passive or change-resistant individuals, adaptive individuals are more likely to make the necessary adjustments to fit into their environment when needed (Fuller et al., 2018). Thus, such a constant tendency to engage in behaviors of adaptation is argued to result in more learning experiences in dealing with various forms of change. Given the urgent, ambiguous, and dynamic nature of crises (Pearson and Clair, 1998; Boin et al., 2005; Mumford et al., 2007), being able to quickly adapt to the imposed situation is a critical resource for any leader (Hadley et al., 2011). Therefore, managers with an adaptive personality are more likely to have the confidence to lead during a crisis as they tend to have the necessary experience to back it up. In other words, we argue that adaptive managers are more likely to believe in their capacity to accurately assess the available information at the time of the crisis and make/recommend the necessary adjustments. Thus, we hypothesize the following:

*Hypothesis 1. Adaptive personality will be positively related to crisis leader self-efficacy*.

### Crisis Leader Self-Efficacy and Leader Motivation During the COVID-19 Crisis

The COVID-19 crisis has established a “new normal” for almost everyone due to its unprecedented far-reaching impact and the needed joint and collective effort by nations, governments, communities, and industries to overcome it (Maragakis, 2020; Mull, 2020; Solomon, 2020). The rapid spread of COVID-19 has only emphasized the need for evolving, adaptive countermeasures to keep up with such volatility, resulting in an environment of ambiguity, complexity, and dynamism that has affected numerous sectors (Chong et al., 2020; Djeebet, 2020; Evans, 2020; Lim-Lange, 2020). The question then becomes “what would increase managers' motivation to lead during the COVID-19 crisis?” Chan and Drasgow (2001) introduced the construct of motivation to lead and defined it as a “construct that affects a leader's or leader-to-be's decisions to assume leadership training, roles, and responsibilities and that affect his or her intensity of effort at leading and persistence as a leader” (p. 482). One core mechanism discussed by the authors regarding enhancing one's motivation to lead is one's beliefs of self-efficacy (Mitchell and Beach, 1976; Mitchell, 1980; Bandura, 1986; Chan and Drasgow, 2001).

Drawing insight from the COR theory, personal resources such as crisis leader self-efficacy are more likely to play a vital role in how managers respond to crises such as COVID-19 (Hobfoll, 1989; Hadley et al., 2011). More specifically, we argue that managers tend to be faced with two options in terms of reacting to the COVID-19 crisis: (1) withdraw from the leadership role and/or responsibilities in a last attempt to save their current resources or (2) utilize their current resources to offset the resource loss associated with COVID-19 and probably compensate for the loss. The COR theory suggests that one's decision to withdraw or tackle a stressful situation will depend on one current pool of resources and its relevance to the situation (Hobfoll et al., 2018). A previous study empirically demonstrated that self-efficacy is positively related to motivation (Çetin and Aşkun, 2018). Therefore, we argue that a manager who is confident in their capability to lead during times of crisis is more likely to be motivated to lead during the COVID-19 crisis instead of vulnerably suffering the losses as they see themselves having the necessary resources to turn the tide in their favor. Thus, we hypothesize the following:

*Hypothesis 2. Crisis leader self-efficacy will be positively related to motivation to lead during the COVID-19 crisis*.

Furthermore, integrating the SCT and COR theory, we argue for adaptive managers' potential to be motivated to lead during the COVID-19 crisis due to their beliefs of self-efficacy to lead during a crisis. More specifically, due to the tendency of adaptive managers to welcome change and modify their ways when needed (Fuller et al., 2018), they are more likely to have accumulated a wealth of knowledge and experience in adapting to situations of ambiguity, complexity, and dynamism, resulting in them experiencing high levels of self-efficacy to lead in such situations, including crises. As a result, such high levels of crisis leader self-efficacy from years of experience are argued to enhance the manager's motivation to lead during an actual crisis, such as that of COVID-19. Thus, we hypothesize the following:

*Hypothesis 3. Crisis leader self-efficacy will mediate the relationship between adaptive personality and motivation to lead during the COVID-19 crisis*.

### Adaptive Performance as a Form of Effective Leadership During the COVID-19 Crisis

The COVID-19 crisis has brought about numerous changes to the structure of many organizations as well as their job designs (Foss, 2020a,b; Seetharaman, 2020). Given the sudden, imposed nature of the COVID-19 crisis, one effective form of action that managers are apt to take in response to the accompanied change in job requirements is to demonstrate adaptive performance (Allworth and Hesketh, 1999; Griffin et al., 2007; Jundt et al., 2015). Adaptive performance has been defined as “task-performance-directed behaviors individuals enact in response to or anticipation of changes relevant to job-related tasks” (Jundt et al., 2015, pp. 54–55). In the workplace, adaptive performance tends to be exhibited when individuals need to adjust their knowledge, skills, and abilities to “adopt new roles, acquire new skills, or. modify existing work behaviors” (Chan, 2000, p. 2) such that they are able to maintain their level of performance or reduce any performance loss during instances of change. Furthermore, adaptive performance can be both anticipatory and/or reactive such that it demonstrates not only behaviors of learning and preparation for anticipated changes but also react to ones that have already occurred (Jundt et al., 2015). Adaptive performance also includes cognitive and/or skill-based adaptations as well as interpersonal and structural ones as long as the individual and the organization can minimize the losses associated with change and reap its benefits when possible (Jundt et al., 2015).

Managers who exhibit adaptive performance tend to (1) handle emergencies and stress by remaining calm during times of difficulty and ambiguity while quickly analyzing options for dealing with such times, (2) engage in creative problem-solving by employing and generating new, unique ideas, (3) always be on the lookout for information that will enhance their learning and improve their work methods, and (4) demonstrate interpersonal flexibility by welcoming other people's views and cooperating with them (Pulakos et al., 2000; Charbonnier-Voirin et al., 2010). These types of behaviors are more likely to minimize the resource loss associated with the COVID-19 crisis, rendering these behaviors an effective form of leadership during such a time. Therefore, managers who are motivated to lead during the COVID-19 crisis are more likely to dedicate their effort and time in a way that enhances their well-being, the well-being of their team, and the success of the organization as a whole; in other words, they are more likely to engage in adaptive performance as a behavioral manifestation of such motivation. Pulakos et al. (2002) investigated the taxonomy of adaptive performance using supervisor ratings of their employees' performance and found that self-efficacy and motivation are significant predictors of adaptive performance. Thus, in the context of COVID-19, we hypothesize the following:

*Hypothesis 4. Motivation to lead the during the COVID-19 crisis will be positively related to adaptive performance during the COVID-19 crisis*.

Building on the previous arguments and integrating the SCT and COR theory (Bandura, 1986; Hobfoll, 1989), managers are more likely to engage in adaptive performance if they believe in their capability to make a change in response to a situation; otherwise, it will seem like an unworthy investment of energy and time, which is also seen as a source of loss (Halbesleben and Buckley, 2004; Hobfoll et al., 2018). This also relates to Lazarus and Folkman (1984) concept of secondary appraisal, which states that the type of coping strategy an individual implements depends on the individual's appraisal of whether they have the necessary resources and ability to cope with the situation. Therefore, managers that have crisis leader self-efficacy are more likely to be motivated to lead during the COVID-19 and demonstrate such capability to reduce the losses associated with such a crisis by engaging in adaptive performance. Furthermore, such managers are more likely to have such high beliefs of self-efficacy and motivation to lead during the COVID-19 crisis because of their past experience in dealing with similar ambiguous, dynamic, and/or challenging situations, which is the case for managers with adaptive personality (Pulakos et al., 2002). Thus, we hypothesize the following:

*Hypothesis 5. Motivation to lead during the COVID-19 crisis will mediate the relationship between crisis leader self-efficacy and adaptive performance during the COVID-19 crisis*.

*Hypothesis 6. Crisis leader self-efficacy and motivation to lead during the COVID-19 crisis will sequentially mediate the relationship between adaptive personality and adaptive performance during the COVID-19 crisis*.

## Methods

### Participants

Online surveys were randomly distributed among full-time managers in public, private, and charitable sectors in Saudi Arabia through multiple channels (e.g., social media outlets, training courses, and executive MBA courses) with instructions emphasizing the targeted population. Furthermore, the data were collected during the summer of 2020 (May–August) to reflect the targeted context of the COVID-19 crisis. We asked every participant to state whether they currently work in a full-time managerial position or not at the time of taking the survey, thereby filtering out those who do not. An initial sample size of around 196 was collected. Utilizing the listwise-deletion method of missing data and deleting responses that failed the attention checks (e.g., “we appreciate your attention, please choose “strongly disagree” for this item”), the final sample size was 116. This method was used because the authors expect the data to be missing completely at random and have sufficient statistical power (Newman, 2014). The sample size adheres to the recommended ratio of 15 observations per independent variable and the preferred sample size of 90 observations to run the analysis in this study, as suggested by Hair et al. (2018). Furthermore, to have a power of 0.80 (i.e., 1-β), resulting in limited a possibility of a type 2 error of 0.20 (i.e., β), with an anticipated medium effect size of.15 at an α equal to 0.05, we collected a sample size that is larger than the minimum recommended sample size of 97 based on Cohen (1992). Thirty-two percent of the respondents were female and their ages ranged from 25 to 74 years old with an average of 43 years. Respondents' work experience ranged from 2 to 45 years with an average of 18 years. The sample characteristics and basic descriptive analysis are provided in Table 1.

**Table 1:** Sample characteristics.

**Variables**	**Frequency** ** (*N* = 116)**	**Percentage (%)**
**Gender**		
Male	79	68
Female	37	32
**Education**		
No schooling	1	1
High school graduate	2	2
Some college credits	1	1
Associate degree	4	3
Bachelor's degree	30	26
Master's degree	30	26
Ph.D. Degree	48	41
**Sector**		
Public	77	67
Private	35	30
Charity	4	3
**Organizational experience**		
6–18 months	5	4
18 months to 3 years	14	12
3–5 years	16	14
Over 5 years	81	70

### Measurement

In addition to utilizing the English versions of the used measures, we created Arabic versions of all the used measures following Brislin (1980) back-translation procedures. All items were measured on a five-point Likert-type scale where 1 = strongly disagree and 5 = strongly agree. *Adaptive personality* was measured using the 14-item scale developed by Fuller et al. (2018). We asked the participants to indicate to what extent they agree or disagree with a set of statements regarding their trait characteristics in general. An example item includes “I am flexible when it comes to making changes.” *Crisis leader self-efficacy* was measured using the eight-item scale developed by Hadley et al. (2011). We asked the participants to indicate to what extent they agree or disagree with a set of statements regarding their self-efficacy to lead *during a crisis*. Item number 3 of the original scale was removed due to it having a low factor loading. An example item includes “I can anticipate the political and interpersonal ramifications of my decisions and actions.” *Motivation to lead during the COVID-19 crisis* was measured using an adapted version of the eight-item scale of Chan and Drasgow (2001) general measure of motivation to lead, which is similar to that of Hadley et al. (2011) in order to reflect the context of the COVID-19 crisis. In doing so, we reduced the total number of items from 27 in the original scale to the eight items that are most relevant to the COVID-19 crisis. We asked the participants to indicate to what extent they agree or disagree with a set of statements regarding their motivation to lead *during the COVID-19 crisis*. An example item includes “I am the type of person who likes to be in charge of others.” *Adaptive performance* was measured using the 19-item scale developed by Charbonnier-Voirin et al. (2010) based on Pulakos et al. (2000) conceptualization of adaptive performance. We asked the participants to indicate to what extent they agree or disagree with a set of statements regarding their performance *during the COVID-19 crisis*. An example item includes “I quickly take effective action to solve the problem.” *Gender, age*, and *organizational tenure* were used as control variables as these variables have been found to be associated with an individual's adaptive performance (Pulakos et al., 2000).

### Analysis

Hierarchical multiple regression analysis was used to assess the direct effect among adaptive personality, crisis leader self-efficacy, motivation to lead during the COVID-19 crisis, and adaptive performance during the COVID-19 crisis. To assess the mediation effect, a test was conducted via the PROCESS macro (v3.5) using SPSS 27 software with the bootstrap sampling method (sample size = 5,000), as recommended by Hayes (2013). The bootstrap sampling method was used to generate asymmetric confidence intervals (CIs) for the mediating effect.

## Results

Harman's single factor test (Harman, 1967) was conducted to check for the existence of Common Method Bias (CMB). For this test, a substantial amount of CMB is present if a single factor emerges from the factor analysis, or one general factor accounts for most of the covariance among the variables (Podsakoff et al., 2012). Principal component analysis with varimax rotation on the questionnaire items revealed the existence of 14 distinctive factors with eigenvalues >1.0. These factors accounted for 70.88% of the total variance. Moreover, the first (and largest) factor accounted for 31.19% of the total variance, which is significantly <50% (i.e., the minimum threshold for influential CMB as per Harman's single factor test; Podsakoff et al., 2012). Since more than one factor emerged and no general factor accounted for the majority of the total variance, concerns of CMB were minimized and CMB was less likely to have significantly confounded the results of this study (Podsakoff et al., 2003). Also, the correlations among the study variables were examined to detect if they showed any sign of inflation (Spector, 2006). The correlations among the observable variables were within the acceptable range except for adaptive personality and performance, which is justifiable because they are closely related constructs, yet distinctive. This empirical evidence together with the consistency of the findings with the theoretical argument and previous research should alleviate any concerns related to CMB.

Table 2 provides the means, standard deviations, correlation coefficients, and reliabilities of the study variables. All the internal consistency reliabilities of the study variables were acceptable for research purposes (above 0.70; Hair et al., 2018). Adaptive personality was found to be positively correlated with crisis leader self-efficacy (*r* = 0.58, *p* < 0.01), motivation to lead during the COVID-19 crisis (*r* = 0.62, *p* < 0.01), and adaptive performance during the COVID-19 crisis (*r* = 0.78, *p* < 0.01). Similarly, crisis leader self-efficacy was positively correlated with motivation to lead during the COVID-19 crisis (*r* = 0.61, *p* < 0.01) and adaptive performance during the COVID-19 crisis (*r* = 0.64, *p* < 0.01), respectively. Lastly, motivation to lead during the COVID-19 crisis was positively correlated with adaptive performance during the COVID-19 crisis (*r* = 0.65, *p* < 0.01).

**Table 2:** Means, standard deviations, correlations, and reliabilities (*N* = 116).

**Variables**	***M***	***SD***	**1**	**2**	**3**	**4**	**5**	**6**	**7**
1. Adaptive personality	4.31	0.44	(0.90)						
2. Crisis leader self-efficacy	4.01	0.48	0.58^**^	(0.78)					
3. Motivation to lead	4.00	0.58	0.62^**^	0.61^**^	(0.74)				
4. Adaptive performance	4.27	0.42	0.78^**^	0.64^**^	0.65^**^	(0.88)			
5. Age	43	8.53	−0.09	−0.04	0.06	−0.06	–		
6. Gender	0.32	0.47	0.21^*^	0.14	0.06	0.23^*^	0.08	–	
7. Organizational tenure	4.50	0.87	−0.03	−0.05	0.03	0.00	0.37^**^	0.12	–

*Reliabilities in (). Gender: 0 = Male, 1 = Female; Motivation to Lead and Adaptive Performance reflect the levels of motivation and performance during the COVID-19 crisis, respectively*.

** *p < 0.01*.

* *p < 0.05*.

Table 3 summarizes the regression results for hypotheses 1, 2, and 4. All of the models were not susceptible to multicollinearity as they had tolerance values well above 0.2 and Variance Inflation Factors (VIF) well below 5 (Bowerman and O'Connell, 1990). Hypothesis 1 was supported as adaptive personality positively predicted crisis leader self-efficacy in Model 2 (*b* = 0.62, *p* < 0.01). Hypothesis 2 was also supported as crisis leader self-efficacy positively predicted motivation to lead during the COVID-19 crisis in Model 4 (*b* = 0.75, *p* < 0.01). Lastly, hypothesis 4 was supported as motivation to lead during the COVID-19 crisis positively predicted adaptive performance during the COVID-19 crisis in Model 7 (*b* = 0.47, *p* < 0.01).

**Table 3:** Summary of the hierarchical regression results (Unstandardized coefficients; *N* = 116).

	**Crisis leader self-efficacy**		**Motivation to lead**			**Adaptive performance**			
**Variables**	**Model 1**	**Model 2**	**Model 3**	**Model 4**	**Model 5**	**Model 6**	**Model 7**	**Model 8**	**Model 9**
Intercept	4.17^**^	1.37^**^	3.81^**^	0.70	−0.61	4.36^**^	2.58^**^	1.90^**^	0.81^**^
Age	0.00	0.00	0.00	0.01	0.01	0.00	0.00	0.00	0.00
Gender	0.16	0.03	0.08	−0.05	−0.12	0.21^**^	0.18^**^	0.14^*^	0.07
Organizational tenure	−0.03	−0.02	0.01	0.03	0.03	0.00	0.00	0.01	0.01
Adaptive personality		0.62^**^			0.55^**^				0.51^**^
Crisis leader self-efficacy				0.75^**^	0.46^**^			0.31^**^	0.17^**^
Motivation to lead							0.47^**^	0.31^**^	0.14^*^
*R*^2^	0.03	0.34	0.01	0.38	0.50	0.06	0.47	0.54	0.70
Δ*R*^2^	0.00	0.31	0.00	0.37	0.12	0.00	0.41	0.07	0.16
*F*	0.98	14.22^**^	0.25	17.09^**^	21.75^**^	2.32	24.16^**^	25.70^**^	40.92
*df*	112	111	112	111	110	112	111	110	109

*Gender: 0 = Male, 1 = Female; Motivation to Lead and Adaptive Performance reflect the levels of motivation and performance during the COVID-19 crisis, respectively*.

** *p < 0.01*.

* *p < 0.05*.

**Figure 2:**
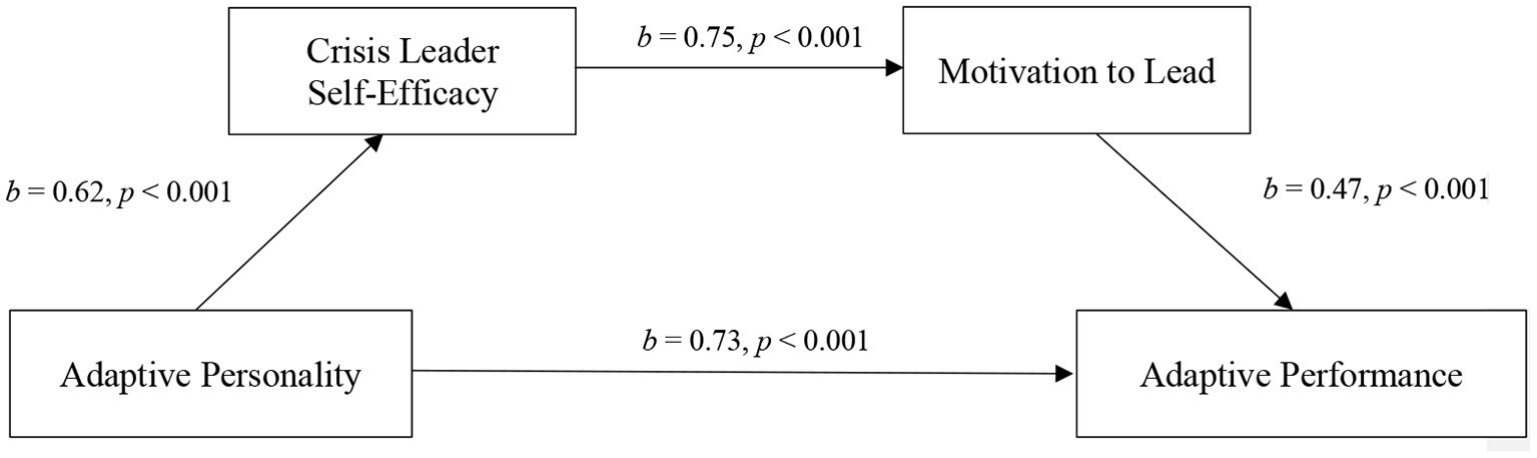
The unstandardized coefficients for the indirect relationship between adaptive personality and adaptive performance through crisis leader self-efficacy and motivation to lead during the COVID-19 crisis (*N* = 116). Total effect, *b* = 0.73, *SE* = 0.06, *p* = 0.001; Direct effect*, b* = 0.51, *SE* = 0.07, *p* = 0.001; Total Indirect effect, *b* = 0.22, *SE* = 0.06, 95% BCa CI [0.10, 0.36]; Indirect effect through crisis leader self-efficacy, *b* = 0.11, *SE* = 0.06, 95% BCa CI [0.01, 0.24]; Indirect effect through motivation to lead, *b* = 0.11, *SE* = 0.05 95% BCa CI [0.01, 0.22]. Motivation to lead and adaptive performance reflect the levels of motivation and performance during the COVID-19 crisis, respectively.

To test hypotheses 3, 5, and 6, Hayes (2013) PROCESS add-on was utilized. The results indicate that the indirect effect of adaptive personality on motivation to lead during the COVID-19 crisis through crisis leader self-efficacy was statistically significant (*b* = 0.29, SE = 0.09, 95% BCa CI [0.14, 0.49]), supporting hypothesis 3. Furthermore, the results show that the indirect effect of crisis leader self-efficacy on adaptive performance through motivation to lead during the COVID-19 crisis was statistically significant (*b* = 0.23, SE = 0.06, 95% BCa CI [0.11, 0.35]), supporting hypothesis 5. Lastly, the results show that the indirect effect of adaptive personality on adaptive performance through crisis leader self-efficacy and motivation to lead during the COVID-19 crisis was statistically significant (*b* = 0.22, SE = 0.06, 95% BCa CI [0.10, 0.36]), supporting the full serial mediation as argued for in hypothesis 6 (see Figure 2).

## Discussion

This study investigates the role personality plays in the emergence of effective leaders during the COVID-19 crisis. More specifically, it examines the effect of the newly developed construct of adaptive personality on full-time managers' adaptive performance in Saudi Arabia during the COVID-19 crisis. Furthermore, this study examines crisis leader self-efficacy and motivation to lead during the COVID-19 crisis as two sequential, explanatory mechanisms between adaptive personality and adaptive performance during the COVID-19 crisis based on Hadley et al. (2011) theoretical framework. The findings indicate that managers with an adaptive personality are more likely to have increased levels of self-efficacy to lead during the times of a crisis, which supports previous research that has emphasized the importance of personality in the development of one's confidence to perform (Larson and Borgen, 2006; Fuller and Marler, 2009; Li et al., 2017). The findings also indicate that crisis leader self-efficacy was found to be significantly related to motivation to lead during the COVID-19 crisis, suggesting that managers who have high beliefs regarding their capability to lead in any crisis are more likely to be motivated to lead during the COVID-19 crisis. Furthermore, those managers were found to be more likely to manifest such motivation by demonstrating adaptive performance given its relevance during times of much needed adaptivity due to the sudden, imposed organizational changes (Jundt et al., 2015; Strauss et al., 2015; Park and Park, 2019).

### Theoretical Implications

This study has multiple theoretical contributions. First, it contributes to the scholars' work on adaptivity such as that of Hirschi et al. (2015) and Rudolph et al. (2017) by finding support for the reliability and predictive validity of adaptive personality, a newly developed construct by Fuller et al. (2018) and Baard et al. (2014). More specifically, the construct of adaptive personality in this study follows and provides empirical evidence for the conceptual framework discussed in Rudolph et al. (2017) based on the career construction model of adaptation (Savickas, 2005, 2013; Savickas et al., 2009; Savickas and Porfeli, 2012) such that adaptivity as a trait (adaptive personality) tends to result in adaptation results (adaptive performance) through adapting responses (e.g., self-efficacy).

Second, drawing insight from the SCT and COR theory, this study finds support for the role of individual differences in influencing the leader's behavior through self-efficacy and motivation to lead as argued for by the theoretical framework developed by Chan and Drasgow (2001). Taking the context of crisis into consideration, this study, therefore, provides empirical evidence for Hadley et al. (2011) adopted theoretical framework, based on Chan and Drasgow (2001) framework, such that individual characteristics tend to affect one's crisis leader self-efficacy, motivation to lead during a crisis, and, as a result, their performance during the crisis.

Third, this study contributes to the crisis management literature by investigating the role of individual differences in influencing one's coping outcomes during the COVID-19 crisis in Saudi Arabia, thereby expanding the findings to new contexts. For instance, a study by Zacher and Rudolph (2021) collected data from 979 individuals in Germany and found that individual differences in life satisfaction, positive affect, and negative affect result in different types and levels of coping strategies during the COVID-19 crisis (e.g., controllability appraisals, positive reframing, using emotional support, self-blame). Furthermore, utilizing a sample of 408 doctors and nurses in Wuhan City, China, another study by Yi-Feng Chen et al. (2021) found that proactive personality tends to influence one's performance, resilience, and thriving through strengths use during the COVID-19 crisis. Such findings from multiple countries emphasize the importance of individual differences and its persistence in coping with the COVID-19 crisis.

### Practical Implications

This study also offers multiple practical implications regarding crisis management, especially during the current times of the COVID-19 crisis. First, it recommends that organizations recruit and hire managers with adaptive personality due to their increased adaptive performance during crises such as that of COVID-19, with their increased motivation and confidence to lead during a crisis. Second, although personality traits are relatively stable, they are not completely static (Robins et al., 2001; Damian et al., 2019); therefore, current managers should be assigned to attend training programs that enhance their adaptivity and adaptive behaviors to be better able to handle the COVID-19 crisis and other similar situations (Aguinis and Kraiger, 2009). Third, organizations should provide a culture of adaptivity for the adaptive managers to thrive in and eliminate any factors that might hinder the manifestation of their motivation as adaptive performance (Schein, 2010).

### Limitations and Future Directions

This study has limitations like any other. First, due to the nature of the studied constructs, the data collection was based on a self-report design where the managers responded to statements regarding their own personality, beliefs, motivation, and performance, which might raise small issues of CMB (Podsakoff et al., 2012). Although (1) a study by Fuller et al. (2016) indicate that CMB needs to be present in high levels before it becomes influential in single-source studies and (2) the results of this study regarding the Harman single factor test, correlational analysis, and VIFs mitigate any concerns relating to CMB (Harman, 1967; Kock, 2015), future research should further control for CMB when attempting to replicate the findings of the study using other techniques (e.g., the correlational marker technique, the CFA marker technique; Lindell and Whitney, 2001; Williams et al., 2010, respectively). Second, due to the self-report nature of the study, subjective measures of the leaders' adaptive performance were collected. Although this might raise some concerns regarding the validity of the outcome, Janssen (2001) noted that subjective measures of performance are also as effective as other-rated performance measures as objective measures of performance are more likely to result in idiosyncratic interpretations and are likely to vary across different raters. Third, this study utilized a cross-sectional design to collect the data by collecting all the data at a single point in time, which might raise concerns regarding the hypothesized relationships' temporal precedence (Bowen and Wiersema, 1999). Thus, future research should utilize a longitudinal or a time-wave design when replicating this study through collecting the data at multiple points in time (Ployhart and Vandenberg, 2010).

The findings of this study also shed light on potential future research avenues. First, although the importance of adaptive performance during crises has been noted (Pulakos et al., 2000), future research would benefit from examining how the adaptive performance of managers, in the context of crises, relates to other more distal forms of performance and leadership effectiveness measures (e.g., creative performance, contextual performance, role performance, department performance; Katz and Kahn, 1978; Campbell, 1990; Borman and Motowidlo, 1993; Harari et al., 2016). Another future research avenue involves investigating other explanatory mechanisms through which a manager's adaptive personality affects their adaptive performance. For instance, the concept of resilience can act as such explanatory mechanism as those who are resilient tend to have basic abilities to adapt to adverse events based on their individual, unit, family, and community resources (Masten et al., 2009; Britt et al., 2016). In other words, adaptive managers are more likely to have increased levels of individual resources and, as a result, resilience as they tend to have faced and adapted to challenging situations throughout their life, rendering them more able to adapt to such situations in the future, and thus, demonstrate it through adaptive performance. Lastly, future research can investigate the influence of the COVID-19 crisis as a moderator of the investigated relationships in this study, and how such a crisis might have a different, unique impact compared to other types/forms of crises (e.g., due to lockdown or isolation) on outcomes such as utilization of online resources and/or teleworking (Belzunegui-Eraso and Erro-Garcés, 2020; Contreras et al., 2020).

## Conclusion

In this paper, we investigate a newly developed measure of adaptive personality as a potential antecedent to what constitutes effective leadership during the unprecedented COVID-19 crisis. More specifically, we investigate crisis leader self-efficacy and motivation to lead during the COVID-19 crisis as two mediators that help explain the relationship between adaptive personality and adaptive performance during the crisis. The results suggest that adaptive managers are more likely to be more confident in themselves to lead during a crisis and, thus, be more motivated to lead during the actual COVID-19 crisis. As a result, they are more likely to invest more time and energy to adapt to the situation at hand through behaviors such as effective handling of emergencies and work stress, creative problem solving, constant learning, and interpersonal adaptability, rendering such managers as an invaluable asset to any department or organization.

## Data Availability Statement

The raw data supporting the conclusions of this article will be made available by the authors, without undue reservation.

## Ethics Statement

The studies involving human participants were reviewed and approved by Deanship of Scientific Research, King Abdulaziz University. The patients/participants provided their written informed consent to participate in this study.

## Author Contributions

ABaj: conceptualization, wrote–original draft, wrote–review and editing, and visualization. SBaj: methodology, formal analysis, investigation, wrote–review and editing, funding acquisition, and project administration. MA, ABas, and SBas: funding acquisition, resources, and conceptualization. All authors contributed to the article and approved the submitted version.

## Conflict of Interest

The authors declare that the research was conducted in the absence of any commercial or financial relationships that could be construed as a potential conflict of interest.
